# “When Music Speaks”: Auditory Cortex Morphology as a Neuroanatomical Marker of Language Aptitude and Musicality

**DOI:** 10.3389/fpsyg.2017.02096

**Published:** 2017-12-01

**Authors:** Sabrina Turker, Susanne M. Reiterer, Annemarie Seither-Preisler, Peter Schneider

**Affiliations:** ^1^Centre for Systematic Musicology, University of Graz, Graz, Austria; ^2^Department of Linguistics, University of Vienna, Vienna, Austria; ^3^BioTechMed-Graz, Graz, Austria; ^4^Section of Biomagnetism, Department of Neurology, University Hospital Heidelberg, Heidelberg, Germany; ^5^Division of Neuroradiology, University Hospital Heidelberg, Heidelberg, Germany

**Keywords:** neuroanatomical correlates, language aptitude, musicality, working memory, auditory cortex morphology, Heschl’s gyrus

## Abstract

Recent research has shown that the morphology of certain brain regions may indeed correlate with a number of cognitive skills such as musicality or language ability. The main aim of the present study was to explore the extent to which foreign language aptitude, in particular phonetic coding ability, is influenced by the morphology of Heschl’s gyrus (HG; auditory cortex), working memory capacity, and musical ability. In this study, the auditory cortices of German-speaking individuals (*N* = 30; 13 males/17 females; aged 20–40 years) with high and low scores in a number of language aptitude tests were compared. The subjects’ language aptitude was measured by three different tests, namely a Hindi speech imitation task (phonetic coding ability), an English pronunciation assessment, and the Modern Language Aptitude Test (MLAT). Furthermore, working memory capacity and musical ability were assessed to reveal their relationship with foreign language aptitude. On the behavioral level, significant correlations were found between phonetic coding ability, English pronunciation skills, musical experience, and language aptitude as measured by the MLAT. Parts of all three tests measuring language aptitude correlated positively and significantly with each other, supporting their validity for measuring components of language aptitude. Remarkably, the number of instruments played by subjects showed significant correlations with all language aptitude measures and musicality, whereas, the number of foreign languages did not show any correlations. With regard to the neuroanatomy of auditory cortex, adults with very high scores in the Hindi testing and the musicality test (AMMA) demonstrated a clear predominance of complete posterior HG duplications in the right hemisphere. This may reignite the discussion of the importance of the right hemisphere for language processing, especially when linked or common resources are involved, such as the inter-dependency between phonetic and musical aptitude.

## Introduction

There has always been a fascination with the simple fact that some individuals are strikingly better at doing something, e.g., playing an instrument, singing, or learning a foreign language. It is said that these individuals possess a certain aptitude, i.e., a potential for developing exceptional ability (Gagné, 1995, 2005; Faulkner, 2003; Nardo and Reiterer, 2009; Al-Shabatat, 2013; Stern and Neubauer, 2013). According to Gagné (1995, 2005), aptitude designates the innate property that develops into a certain skill, which is then termed talent (Stern and Neubauer, 2013; Deiglmayr et al., 2017). Individuals with a high aptitude for something mostly put little effort into acquiring a certain skill and need far less time and practice to reach a high achievement or proficiency level in comparison to age-matched peers (Carroll, 1958, 1962, 1990). The concept of language aptitude has gained considerable momentum in the past decades and research has shown that various factors contribute to the overall achievement and proficiency of skills, e.g., environmental influences, personality traits, motivation and other abilities such as musicality or working memory (Ganschow and Sparks, 1995; Dörnyei, 1998, 2006; Sparks and Ganschow, 2001; Brown, 2006; Biedroń and Szczepaniak, 2009; Rota and Reiterer, 2009; Biedroń, 2011a,b, 2012; Sparks et al., 2011; Wen, 2012, 2016; Christiner and Reiterer, 2013; Granena and Long, 2013; Dörnyei and Ryan, 2015; Li, 2015, 2016; Biedroń and Pawlak, 2016a,b; Singleton, 2017; Wen et al., 2017).

Language aptitude is a vague concept challenging to grasp and even more difficult to measure accurately (Li, 2015). In the 1st years after the birth of language aptitude research, it was regarded as an exceptional ability that facilitates foreign language learning in terms that individuals learn a language very quickly and with little effort (Carroll, 1958, 1962, 1973, 1990; Stansfield and Reed, 2004). For a long time, language aptitude was thus defined by the rate of acquisition at which an unknown language was learned. More recent definitions (Robinson, 2005) describe it as the strength of an individual with respect to cognitive abilities especially drawn upon during the learning of foreign languages. In the past years, the focus of foreign language aptitude research has shifted more toward formerly neglected issues, such as the influence and importance of inter-individual differences (Skehan, 1986, 2002; Spolsky, 1995; Dörnyei, 1998, 2006; Robinson, 2001, 2002, 2012; Dörnyei and Skehan, 2003; Biedroń, 2015; Dörnyei and Ryan, 2015; Wen et al., 2017). The four major components of language aptitude claimed by Carroll (1958, 1962), namely (1) Phonetic Coding Ability, (2) Grammatical Sensitivity, (3) Inductive Language Learning Ability, and (4) Rote Learning Ability, are still upheld nowadays. Still, some theoretical advancements have been made and it has been agreed that inductive language learning ability and grammatical sensitivity are most suitably summarized in one category termed language analytic ability (Robinson, 2001, 2002, 2012; Abrahamsson and Hyltenstam, 2008; Kocić, 2010; Biedroń, 2015; Biedroń and Pawlak, 2016a,b; Wen et al., 2017). Moreover, researchers have questioned whether the distinctive components of language aptitude might be more or less influential at different stages and in different contexts of learning (Abrahamsson and Hyltenstam, 2008; Artieda and Muñoz, 2016).

The core factors investigated in this study are working memory, musicality, language aptitude and auditory cortex morphology. Language and music are two inextricably linked concepts that extensively influence one another (Besson and Schön, 2003; Koelsch, 2005; Kraus and Chandrasekaran, 2010; Patel, 2011, 2012; Jäncke, 2012; Chobert and Besson, 2013; Lee and Lin, 2015). A positive correlation between musicality and foreign language aptitude was found in numerous studies, particularly regarding second language pronunciation skills (Schön et al., 2004; Besson et al., 2007; Dogil and Reiterer, 2009; Ludke, 2010; Fonseca-Mora et al., 2011; Christiner and Reiterer, 2013). Whereas Milovanov et al. (2004, 2008, 2009, 2010), Milovanov (2009), Milovanov and Tervaniemi (2011) focused on the successful relationship between musicality and foreign language learning in Finnish native speakers, Vangehuchten et al. (2015) found a significant relationship between English pronunciation skills and musical skills in Spanish native speakers. Dolman and Spring (2014) revealed that excellent skills in specific musical abilities of Japanese learners, such as the discrimination of pitch, loudness, and rhythm, correlate with better pronunciation in the second language (English). Likewise, Slevc and Miyake (2006) found a consistent relationship between musical aptitude and phonological aspects of linguistic ability, but not between syntactic or semantic skills. Similar results were also found for Iranian native speakers (Shabani and Torkeh, 2014). Apart from general musical abilities, singing has also been shown to correlate with foreign language aptitude (Ludke, 2010; Ludke et al., 2014), especially pronunciation aptitude and speech imitation skills (Christiner and Reiterer, 2013, 2015).

Beside the growing interest in musicality as an essential factor for successful foreign language acquisition, the mutual interdependence between working memory (for details, refer to Baddeley and Hitch, 1974, 2000; Baddeley, 2003a,b) and language aptitude has been the focus of most recent research. Due to the strong correlation between the two, some researchers have even gone as far as to claim that working memory capacity is equivalent to language aptitude (Miyake and Friedman, 1998; Sawyer and Ranta, 2001; Wen and Skehan, 2011; Wen, 2016; Wen et al., 2017). Studies on language ability and working memory have confirmed the impact of the latter on numerous linguistic abilities, such as faster and more successful first and second language learning (Ellis and Sinclair, 1996; Miyake and Friedman, 1998; Kormos and Sáfár, 2008; Sáfár and Kormos, 2008; Linck et al., 2013). In other words, those learners who have significantly better working memory skills seem to learn more foreign languages and tend to be more successful (Van den Noort et al., 2006; Biedroń, 2012). However, major issues therein are the differences between specific working memory components, how they can be tested and in how far they relate to the known components of foreign language aptitude (Baddeley, 2003a,b, 2017; Jacquemot and Scott, 2006). Additionally, other studies have questioned the large impact working memory is said to have on language aptitude (Winke, 2013).

While musicality and working memory are mostly treated as clear predictors of foreign language learning ability, the relationship between brain morphology and language aptitude is far from obvious. The processing of language in the human brain has been a subject of investigation in countless studies (for overviews see Friederici, 2009; Price, 2010, 2012; Xiang, 2012), but very few have actually focused on language aptitude or talent (e.g., Golestani et al., 2007, 2011; Reiterer et al., 2011; Xiang et al., 2012; Hu et al., 2013; Kepinska et al., 2017a,b). In this study, we focus on the neuroanatomy of the auditory cortex given its importance for processing speech. The core region containing the primary auditory cortex is Heschl’s gyrus (henceforth always HG), embedded as a transverse gyrus oriented from the insular toward the anterolateral part of the superior temporal lobe. Most humans possess a single or paired HG, the latter in the shape of a common stem or complete posterior duplication (CPD) of HG (Rademacher et al., 1992, 2001; Morosan et al., 2001; Purves et al., 2001; Bear et al., 2006; Hackett, 2009), but HG shows considerable morphological variation between individuals (Heschl, 1878; Galaburda et al., 1978; Rademacher et al., 1992, 2001; Marie et al., 2016), especially in the right hemisphere (Penhune et al., 1996; Schneider et al., 2002, 2005; Seither-Preisler et al., 2014; Serrallach et al., 2016; Benner et al., 2017). Benner et al. (2017) recently found that 90% of musicians had multiplications of HG, mostly on the right side. The right hemisphere has often been claimed to be particularly important for the processing of musical sounds (Zatorre et al., 2002) and less important for speech. Other studies have also suggested that the shape and number of Heschl’s gyri may be an indicator for musical skills and auditory-related developmental disorders such as dyslexia (Schneider et al., 2002, 2005; Warrier et al., 2012; Seither-Preisler et al., 2014; Serrallach et al., 2016; Benner et al., 2017). Seither-Preisler et al. (2014) discovered that a large right HG is associated with high musical aptitude in children. Their longitudinal observations revealed that the gross morphology and gray matter volume of different parts of auditory cortex showed a high inter-individual variability, but remained almost perfectly stable throughout the study, lasting for several years. A regression model showed that this neuroanatomical trait was much stronger associated with measures of musical aptitude than with training-related musical expertise (i.e., the amount of previous training). The authors therefore concluded that an enlarged right HG reflects a high predisposition for music which enhances a child’s intrinsic motivation to learn an instrument. As a consequence, this leads to high musical expertise and boosts learning-induced neural plasticity. It therefore appears worthwhile to explore possible neuroanatomic markers of language aptitude as well.

With regard to language ability, few studies have addressed the significance of specific language-involved regions, such as HG, for language learning. Kepinska (2017) performed a highly appealing study investigating the neural basis of language analytic ability in high and moderate learners. They found that the more skilled learners drew more from neural resources in the right hemisphere (e.g., right angular gyrus, supramarginal gyrus, superior frontal and middle gyrus, and posterior cingulate), in contrast to the less skilled learners. Golestani et al. (2007) found correlations between an abnormal asymmetry of the planum temporale and poor verbal skills. Golestani et al. (2011) reported that the size of the left inferior frontal gyrus (pars opercularis) correlated with the years of experience expert phoneticians had. Additionally, they found that the expert phoneticians more frequently had multiple or split HGs in their core auditory cortex in the left hemisphere. In studies by Wong et al. (2007, 2008), English-speaking adults, who were less successful in learning to incorporate foreign pitch patterns in word identification, exhibited smaller volume of HG in the left hemisphere only.

A widespread belief holds that the left temporal lobe is more tuned for the processing of rapid sound stimuli, which consequently leads to a left-hemispheric dominance for speech processing. This hypothesis assigns information processing in short time windows (e.g., phonemes) to the left and longer time windows (e.g., syllables to intonation profiles) to the right hemisphere (Zatorre et al., 2002; Poeppel, 2003; McGettigan and Scott, 2012). Warrier et al. (2012) also support this by reporting that left HG is of greater importance for varying rates of stimulus change, and right HG for music-relevant functions, such as increasing spectral information. However, McGettigan and Scott (2012) question, whether sensitivity for rapid information is sufficient for efficient speech processing for various reasons, e.g., the identification of differences in the duration of consonants and the encoding of supra-segmental information in speech. Most importantly, they argue that the main issue with this hypothesis and widespread belief is ‘the assumption that access to phoneme representations is the cardinal aspect of speech perception.’

To summarize, the neuroanatomy of HG has been addressed in various studies focusing on sound and speech perception, but only in few studies dealing with language aptitude. We therefore aim at bridging this gap by exploring the importance of the number of HGs in individuals with high and low language learning abilities.

## Materials and Methods

### Subjects

All participants (*N* = 30; 13 male/17 female) were monolingual German native speakers between 20 and 40 years of age (*M* = 26.77, *SD* = 4.95) and had begun acquiring their second language, English, at 10 ± 1 years of age. All participants were right-handed German bachelor/master students or had achieved positions at an institution of higher education. None of the participants showed any medical condition or neurological disorder. The subjects were paid for participation and provided written informed consent before participating in the experiment. The data were analyzed anonymously.

### Language Aptitude Testing

All individuals were classified as high or low aptitude individuals according to two scores, namely an English pronunciation score and a Hindi speech imitation score (both ranging from 0 to 10). The English pronunciation score was based on reading performance of ‘The North Wind and the Sun’ rated by native speakers. In the Hindi task, participants had to repeat four words and four sentences in Hindi, an unknown language to them. The Hindi imitation and the English pronunciation performances were categorized in a similar fashion – both were rated by native speakers according to the correctness and quality of the pronunciation/imitation based on a global intuitive impression rating procedure (the rating procedure is detailed in Reiterer et al., 2011 and Jilka, 2009). The inter-rater reliability was 0.96, i.e., very high, because of the unusually high amount of raters (*N* = 30). Thus, the corresponding scores can be considered as highly reliable. The speakers were recorded on a professional speech recording equipment in the sound-proof basement room of the former phonetics laboratory of the Institute of Natural Language Processing, University of Stuttgart. The native speakers were provided with the speech material online and gave ratings from 0 to 10 on an intuitive Likert-scale-like bar for the quality and ‘nativelikeness’ of the speech material. For the Hindi rating, sound files of Hindi native speakers were added to the rating sample of German speakers (without knowledge of the raters). We used this as additional measure to verify the validity of the rating procedure.

Beside the speech imitation skills (referred to as a measure for phonetic coding ability) and the English pronunciation skills, the Modern Language Aptitude Test (MLAT; Carroll and Sapon, 1957; Ganschow and Sparks, 1995; Dogil and Reiterer, 2009) was used for assessing different components of language aptitude. The parts of the MLAT used were III, IV, and V and the overall total raw score (a combination of the three sub-scores) was further calculated. The three sub-tests provide measures of phonetic coding ability (the ability to differentiate between speech sounds), associative memory (the ability to keep linguistic input in memory and to access this information) and grammatical sensitivity (the ability to understand grammatical relationships and the functions of words in a given context) (Carroll and Sapon, 1957; Carroll, 1958, 1973, 1990); for further details see **Table 1**.

**Table 1:** A description of the different parts of the Modern Language Aptitude Test (MLAT) used in this study (Parts III, IV, and V).

MLAT	Name	Task
Part III	Spelling clues	Sound-symbol association ability and vocabulary knowledge – correct synonyms of disguised words have to be selected (multiple choice).
Part IV	Words in sentences	Grammatical sensitivity – components of sentences have to be identified (grammatical function) and related to elements in other words.
Part V	Paired associates	Associative rote memory – as many words in Kurdish have to be memorized as possible (presented with english translations).

### Musicality Assessment

To assess aptitude in the musical domain, the AMMA test (Gordon, 1980, 2001), a well-established tool for administering musical aptitude, was used. It consists of two parts and has been shown to successfully measure pitch and rhythm perception. The subjects were asked to complete both tasks, (1) a rhythm discrimination task and (2) a pitch discrimination task. Furthermore, a questionnaire was used to specify the number and type of instruments the subjects had learnt in the course of their life.

### Working Memory Capacity

The importance of working memory capacity for language ability has been shown in various studies and different tasks to measure the components of working memory exist. For this study, three types of tests were applied. Subjects had to do a digit span backward, a digit span forward, and non-word span task. The digit span forward test requires subjects to repeat a rising number of digits (starting out with a small number and always adding one per round) in the same order as heard. The digit span backward, in contrast, requires the repetition of heard digits (same procedure as the aforementioned) backward. In the non-word span task participants are asked to repeat non-word syllables in the same order as heard while paying particular attention to the sounds used in these non-words. All participants were given two chances for the same number of digits/non-words, i.e., if the first attempt of repeating a certain amount of digits failed, the subjects heard a different set of the same amount of digits to repeat. Only if both attempts were incorrect, the test was stopped at that point and no points were given. Per correct series, the subjects received one single point.

### Morphometric MRI

**FIGURE 1:**
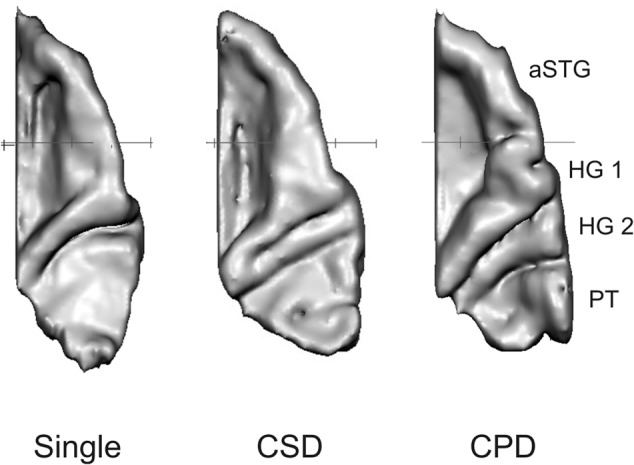
Examples of 3D reconstructions of the three types of HG distinguished in this paper. Examples from the right hemisphere are given (from left to right): (1) single gyrus (SG), (2) common stem duplication (CSD), and (3) complete posterior duplication (CPD). (aSTG anterior superior temporal gyrus; PT planum temporale).

For the neuroanatomical analysis, high-resolution T1-weighted structural magnetic MRI (Siemens, Magnetom SonataVision, 1,5 Tesla, software version: syngo MR 2004A, 176 DICOM slices, sagittal orientation, slice thickness 1 mm) were performed in order to investigate the morphology of auditory cortex in both hemispheres. Three-dimensional gray matter surface reconstructions of the auditory cortex (HG) and the planum temporale (PT) were analyzed using a standardized individual approach. This allows for a closer look at the shape of HG in the subjects’ brains (Schneider et al., 2002, 2009; Seither-Preisler et al., 2014; Serrallach et al., 2016; Benner et al., 2017). Brain Voyager software QX 2.8 (Brain Innovation B.V, Maastricht, Netherlands) was used for the segmentation of the aforementioned auditory-related areas. Pre-processing steps included the adjustment of brain images in contrast and brightness, as well as a correction for inhomogeneity and a rotation in direction of the antero-posterior commissural line. Normalization in stereotactic space (Talairach and Tournoux, 1988) was carried out to arrive at comparable reconstructions. In the process of segmentation, the superior temporal plane, including HG, the anterior superior temporal gyrus and the planum temporale, were segmented into sagittal MRI slices along the lateral fissure using the standard definition of the landmarks of AC. After this semi-manual slice-by-slice segmentation (adapted from Schneider et al., 2005, 2009; also applied by Wengenroth et al., 2010, 2013; Seither-Preisler et al., 2014; Serrallach et al., 2016; Benner et al., 2017), the auditory cortices of all subjects were 3D-reconstructed and the authors compared the shape of HG in each hemisphere. The three categories chosen for categorization were (1) single gyrus (SG), (2) common stem duplication (CSD) and (3) CPD. In **Figure 1** the three types of HG are compared. These categories are in accordance with recent research (Marie et al., 2016; Benner et al., 2017) with the exception of multiple gyri, which were only present in four hemispheres of this study and therefore considered to belong to the CSD group in the case of a *z*-shape (*N* = 2) and to belong to the CPD in the case of more than one CPD (*N* = 2). Lateral HG duplications were considered to be part of the planum temporale and medial duplications (Schneider et al., 2005) to be a sub-form of CSD.

## Results

### Behavioral Results

#### Descriptive Results

First of all, a brief summary of the descriptive results of the variety of tests shall be given. In this study, both the English pronunciation score and the Hindi imitation score are considered as measures for language aptitude. Performance in the English pronunciation task was rather high (*M* = 6.40; *SD* = 1.72) in contrast to the Hindi task. In the Hindi speech imitation task, subjects obtained between 2.72 and 7.74 (*M* = 4.81, *SD* = 1.64) of maximally 10 achievable points, with the mean being much lower than in the English task. As also reported by Dogil and Reiterer (2009), the native speakers that had been mixed into the rating procedure additionally received scores from 8 to 10, i.e., the raters considered scores between 8 and 10 as reflecting native performance. The maximum of points achieved by a German-speaking subject was 7.74, which is strikingly high given that the subject had never been exposed to Hindi. According to speech imitation performance in the Hindi test, subjects with a score below 4 were considered to have very poor skills and were classified as ‘non-talented,’ while subjects with a score above 5 were classified as ‘talented’.

The number of instruments subjects had learnt ranged from zero to three (*M* = 1.23, *SD* = 0.97), with most participants playing one single instrument. In stark contrast, the number of foreign languages acquired ranged from one to nine (*M* = 2.59, *SD* = 1.72), although most subjects had learnt two to three foreign languages.

AMMA tonal results (*M* = 28.72, *SD* = 5.68) were quite similar to AMMA rhythm results (*M* = 31.10, *SD* = 4.61). The total score for the AMMA, subsuming both aforementioned parts, ranged from 42 to 79, i.e., it showed a considerable variability (*M* = 59.8, *SD* = 10.05). In the working memory scales, digit span forward (*M* = 9.59, *SD* = 1.88) and digit span backward (*M* = 8.76, *SD* = 2.13) gave similar results. However, subjects performed better in the forward task. With a range from 6 to 14, some participants showed remarkable results, which were far beyond the norm. The digit span backward scores ranged from 4 to 13, which was still higher than the span for the non-word task (*M* = 7.55, *SD* = 1.74), where subjects scored between 5 and 11 points.

Great variability was found in the MLAT total scores with a range from 49 to 109 points (raw score; *M* = 83.41, *SD* = 14.23), reflecting the large gap between ‘highly gifted’ and ‘poor’ language learners. The MLAT total score summarizes the results of part III (*M* = 36.69, *SD* = 8.62), part IV (*M* = 29.28, *SD* = 5.58) and part V (*M* = 17.31, *SD* = 5.09). The best performance was thus found in part III, measuring phonetic coding ability, and the least successful performance in part V, the vocabulary learning task.

#### Correlational Analysis

As the correlational analyses include multiple variables and comparisons, this may increase the risk of chance findings at a critical *p*-value of 0.05, due to alpha error accumulation. Therefore, the original correlational analysis was complemented by an analysis corrected for multiple comparisons. Similar to the classical Bonferroni correction, the Benjamini–Hochberg procedure we applied is only appropriate for variables independent of each other (McDonald, 2014). Since there is an interdependence between the composite variables AMMA total (consisting of AMMA rhythm and AMMA tonal results), MLAT total (consisting of the three subtests), and an overall pronunciation aptitude score (summarizing the Hindi and English score), this prerequisite was not fulfilled for these variables and they therefore had to be excluded. We used the method of false discovery rate to control for alpha error accumulation in multiple comparisons (Benjamini and Hochberg, 1995). According to the recommendation of the authors, who suggest rates between 0.10 and 0.25 (not to be confounded with regular significance levels, which are much lower), we selected a value of 0.2, corresponding to an acceptable proportion of false discoveries ≤20%. An overview of the correlational results is given in **Table 2**.

**Table 2:** An overview of the correlational analysis performed with SPSS 22.

	Instruments	Languages	Hindi	English	AMMA tonal	AMMA rhythm	MLAT V	MLAT IV	MLAT III	Non-word	Digit backward	Digit forward	Age
Instruments	1	0.159	0.394^∗∗∗^	0.310	0.455^∗∗∗^	0.407^∗∗∗^	–0.021	0.506^∗∗∗^	0.182	0.350	0.282	0.145	0.169
Languages		1	0.003	–0.066	0.218	0.213	0.048	0.217	0.131	0.103	0.273	0.056	0.501^∗∗∗^
Hindi			1	0.390^∗∗∗^	0.284	0.278^∗^	0.356	0.384^∗^	0.290	0.483^∗∗∗^	0.369^∗^	0.447^∗∗∗^	0.248
English				1	0.268	0.393^∗∗∗^	0.333	0.557^∗∗∗^	0.756^∗∗∗^	0.163	0.251	0.195	0.000
AMMA tonal					1	0.911^∗∗∗^	–0.023	0.333	0.243	0.312	0.260	–0.101	0.168
AMMA rhythm						1	–0.009	0.367	0.365	0.313	0.224	0.034	0.042
MLAT V							1	0.278	0.299	0.012	0.165	0.212	–0.028
MLAT IV								1	0.590^∗∗∗^	0.083	0.237	0.246	0.062
MLAT III									1	0.012	0.268	0.320	0.022
Non-word										1	0.335	0.323	0.534^∗∗∗^
Digit backward											1	0.259	0.313
Digit forward												1	–0.145
Age													1

*^∗∗∗^Significant after correction for multiple comparisons (Benjamini–Hochberg procedure). ^∗^Significant at *p* < 0.05 before correction.*

AMMA rhythm and AMMA tonal (*r* = 0.911, *p* = 0.000) showed a very strong relationship with each other. Likewise, different parts of the MLAT correlated significantly with each other, namely MLAT IV and MLAT III (*r* = 0.590, *p* = 0.001), but also with the English pronunciation score (MLAT III and English: *r* = 0.756, *p* = 0.002; MLAT IV and English: *r* = 0.557, *p* = 0.002). MLAT IV and the number of instruments learnt by a subject (*r* = 0.506, *p* = 0.005). Age of participants (ranging from 20 to 40 years) highly correlated with the number of languages (*r* = 0.501, *p* = 0.005) and non-word span (*r* = 0.534, *p* = 0.003). After correction, non-word span and Hindi (*r* = 0.482, *p* = 0.008) and digit span forward and Hindi (*r* = 0.447, *p* = 0.015) still had a strong relationship. The same can be reported for results on Hindi and English tasks (*r* = 0.390, *p* = 0.033), and the Hindi results and number of instruments (*r* = 0.394, *p* = 0.032). Last but not least, AMMA tonal correlated significantly with the number of instruments (*r* = 0.455, *p* = 0.013), whereas AMMA rhythm correlated both with the number of instruments (*r* = 0.407, *p* = 0.028) and the English pronunciation score (*r* = 0.393, *p* = 0.035).

#### Principal Component Analysis

In order to gain insights into the most influential factors underlying performance on the different test scales, we calculated a principle component analysis (PCA). This included the same scales as shown in the correlation matrix of **Table 2**. A pre-analysis of our data showed that the requirements for the application of the method were fulfilled [(a) the determinant as an indicator of multicollinearity, which should be *p* < 0.05, was *p* = 0.002; (b) the Kaiser-Meyer-Olkin criterion as a measure for the suitability of the sample, which should be above *p* = 0.5, was *p* = 0.653; (c) the Bartlett-test for sphericity, which should be significant at least at *p* < 0.05 was significant at *p* < 0.000001]. We used varimax rotation with Kaiser-normalization, which according to the scree-plot yielded a solution with three factors with eigenvalues clearly above 1. The variance explained by the model was 61.2%, which confirms its appropriateness. **Table 3** shows the rotated component matrix with the coefficients of each scale on the three identified components.

**Table 3:** Rotated component matrix with loading coefficients for each scale.

Rotated component matrix			
	**Components**		
	**1**	**2**	**3**
AMMA tonal	**0.918**		
AMMA rhythm	**0.872**		
n° instruments	**0.598**		0.330
n° languages	0.357		
MLAT III		**0.868**	
MLAT IV	0.340	**0.711**	
MLAT V		**0.560**	
English score		**0.829**	
Hindi score		0.312	**0.734**
Non-word span	0.372		**0.729**
Digit span forward			**0.716**
Digit span backward			**0.558**
Extraction method: Principal component analysis. Rotation method: Varimax with Kaiser normalization.			
(a) Rotation converged in 6 iterations.			

*For the sake of clarity, coefficients below 0.3, which signify very small and hence negligible loadings, are not shown. Coefficients above 0.5, which signify particularly relevant contributions, are marked in bold.*

According to the criterion for strong and thus particularly relevant loadings (>0.5), the first component comprises scales related to musicality (AMMA tonal and rhythm scores, number of played instruments), the second component refers to language talent (total English score and parts III, IV, and V of the MLAT) and the third component refers to working memory capacity (digit span forward, digit span backward, non-word span and the Hindi score). Apart from these main findings, there are weaker but still noteworthy loadings (>0.3). These show that the number of instruments played is also associated with the component working memory capacity, while the number of languages spoken is also associated with the component musicality. Moreover, part IV of the MLAT, which measures grammatical sensitivity and is first and foremost a scale of language talent, is also related to musicality. Similarly, non-word span also moderately loads on the component musicality. The total Hindi score, being most strongly related to working memory, also moderately loads on the component language talent.

**FIGURE 2:**
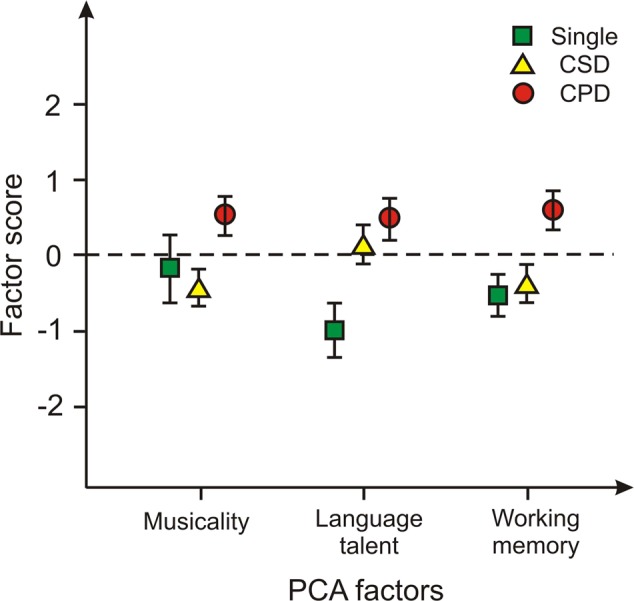
Individual factor scores (positive/negative: above/below average; *M* = 0, *SD* = 1) on each of the three identified PCA components compared for the three types of HG in the right hemisphere. Error bars: SEM (standard error of the mean); Single, single gyrus; CSD, common stem duplication; CPD, complete posterior duplication.

In a next step, the participants’ individual factor scores on each of the three identified components (positive/negative: above/below average; *M* = 0, *SD* = 1) were compared for the three types of right-hemispheric HG morphology (single, common stem, complete duplication). Results are graphically illustrated in **Figure 2**. For all three factors, performance of subjects with complete duplications was highest. Concerning musicality, subjects with a complete duplication were significantly better (*M* = 0.48, *SD* = 0.97) than subjects with common stem morphology [*M* = -0.4, *SD* = 0.76; *t*_(20)_ = -2.4, *p* = 0.027]. With regard to language talent, subjects with a complete duplication were significantly superior (*M* = 0.48, *SD* = 0.80) to subjects with a common stem morphology [*M* = 0.15, *SD* = 0.75; *t*_(16)_ = -2.7, *p* = 0.015] and to subjects with a SG [*M* = -0.99, *SD* = 1.0; *t*_(16)_ = -3.4, *p* = 0.004]. Also with regard to working memory, subjects with a complete duplication were significantly better (*M* = 0.65, *SD* = 0.91) than subjects with a common stem morphology [*M* = -3.2, *SD* = 0.86; *t*_(20)_ = -2.6, *p* = 0.019] and subjects with a SG [*M* = -0.52, *SD* = 0.88; *t*_(16)_ = -2.7, *p* = 0.016].

#### Hindi Speech Imitation Score

A *t*-test based on the distinction between talented and non-talented subjects revealed significant differences between the two groups. This was the case for the number of played instruments [*t*_(28)_ = -2.32, *p* = 0.028], the English pronunciation score [*t*_(28)_ = -2.1, *p* = 0.045], the digit span forward score [*t*_(27)_ = -2.73, *p* = 0.011], the non-word repetition score [*t*_(27)_ = -2.5, *p* = 0.017] and the MLAT total raw score [*t*_(27)_ = -2.27, *p* = 0.032].

Additionally, a linear multiple regression analysis (method: step-wise forward) was performed for the criterion variable Hindi score and the predictors AMMA tonal, AMMA rhythm, English proficiency, MLAT III, MLAT IV, MLAT V, digit span forward, digit span backward, non-word span, number of instruments, and number of learned languages. The model yielded a corrected *R*^2^-value of 0.375, corresponding to an explained variance of 37.5%, and beta-values (relative importance of contributing variables, summing up to 1) of 0.44 for non-word span, 0.31 for the number of instruments played, and 0.25 for part V of the MLAT. In other words, the three most important predictors for the Hindi speech imitation score were performance on non-word span (i.e., working memory capacity), the number of instruments played by an individual and results of MLAT V, measuring grammatical sensitivity. Overall, these three predictors explain 37,5% of the Hindi score, which points to a high explanatory value of the considered variables for phonetic coding ability.

### Results of the Neuroanatomical Analysis

**FIGURE 3:**
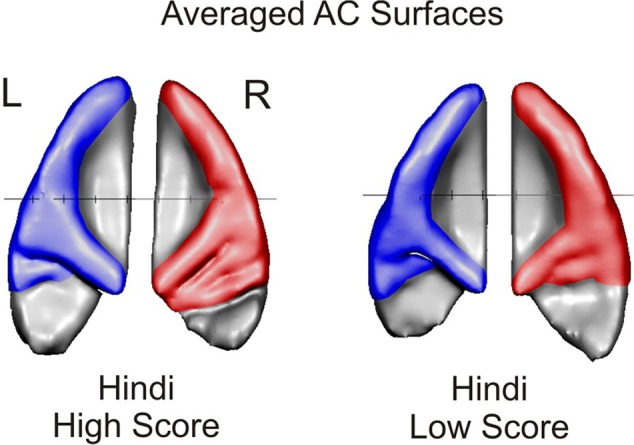
Group-averaged auditory cortex (AC) surfaces of subjects with high Hindi **(left)** and low Hindi scores **(right)**. The predominance of posterior duplications in the right hemisphere (red) of subjects with high Hindi Score is clearly visible from the averaged surface. Subjects with low Hindi score show in the averaged map a lateral HG duplication, which is also visible in the averaged left hemisphere of subjects with high Hindi score.

**FIGURE 4:**
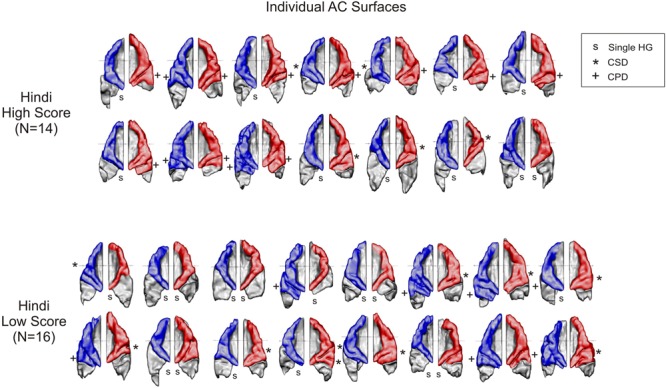
Individual AC surfaces of all 30 subjects, subdivided in subjects with high **(top)** and low **(bottom)** Hindi scores. Subjects of the first group show a clear predominance of CPDs in the right hemisphere. However, trends in the left hemisphere could not be statistically verified. Overall, the incidence of duplications was about twice larger in the right hemisphere (red) as compared to the left hemisphere (blue).

We compared the 3D-reconstructed HGs of both hemispheres in all subjects. First, we categorized all HGs by description (i.e., defining complete duplications and CSDs) as in previous papers (Schneider et al., 2005; Seither-Preisler et al., 2014; Benner et al., 2017). The frequencies of different HG types (altogether *N* = 30) found in our subjects are given in **Table 4**, group-averaged AC surfaces are presented in **Figure 3** and the individual auditory cortices of all subjects are provided in **Figure 4**.

**Table 4:** Frequency of types of HG in right and left hemispheres in subjects with high and low Hindi score.

		RH (high/low)	LH (high/low)
	Total number (%)	30 (100%)	30 (100%)
Types of HG	Single	8 (26%) (1/7)	18 (60%) (9/9)
	CSD	11 (37%) (3/8)	3 (10%) (2/1)
	CPD	11 (37%) (10/1)	9 (30%) (3/6)

*RH, right hemisphere; LH, left hemisphere; CSD, common stem duplication; CPD, complete posterior duplication.*

**Figures 3 and 4** nicely portray the differences found in HG morphology in the right hemisphere. It is clearly evident that individuals with high speech imitation aptitude in the Hindi testing, and also individuals with very high scores in the AMMA testing, showed more CPDs of their HG in the right hemisphere. This means that subjects with excellent scores in the language aptitude and in the AMMA testing have two equally prominent HGs in the right hemisphere, in contrast to those with rather low scores, who possess most frequently single gyri or a CSD.

In order to verify the significance of the described exemplary observations, we performed one-way ANOVAs on the Hindi and AMMA test scores for subjects displaying one of the three following morphological HG characteristics in their right hemisphere: (1) SG, (2) CSD, and (3) CPD (double gyrus; CPD). Furthermore, χ^2^-tests were performed on the frequency distributions of these neuroanatomical characteristics.

**FIGURE 5:**
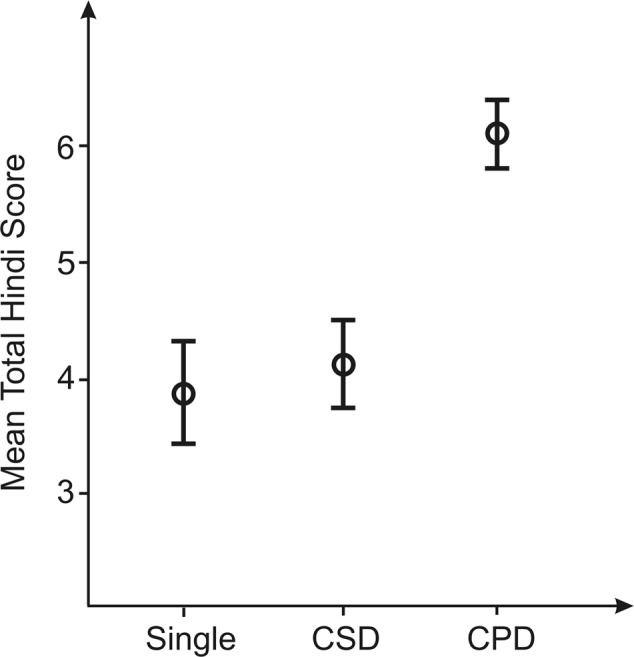
Results of the one-way ANOVA comparing mean total Hindi score (overall range: 0–10) with the three types of HG in the right hemisphere (for a visual presentation, see **Figure 1**). Error bars: SEM (standard error of the mean). Individuals with CPD scored significantly higher in the Hindi testing in comparison to subjects with SG or CSD in the right hemisphere.

A significant group difference was observed for the Hindi speech imitation score [*F*(2,27) = 9.2, *p* < 0.001, $\eta_{p}^{2}$ = 0.41] (**Figure 5**). Subjects with a CPD achieved significantly higher scores (6.1 ± 1.2) than subjects with a SG (3.9 ± 1.4; *p* = 0.002) and subjects with a CSD (4.1 ± 1.4; *p* = 0.004). There was no significant difference in Hindi imitation between individuals with a SG and a CSD. This is also reflected by the fact that among the high performers in the Hindi speech imitation task CPD in the right hemisphere occurred most frequently (71%) while in low performers they occurred most rarely [6%; χ^2^(2) = 14.1, *p* < 0.001).

**FIGURE 6:**
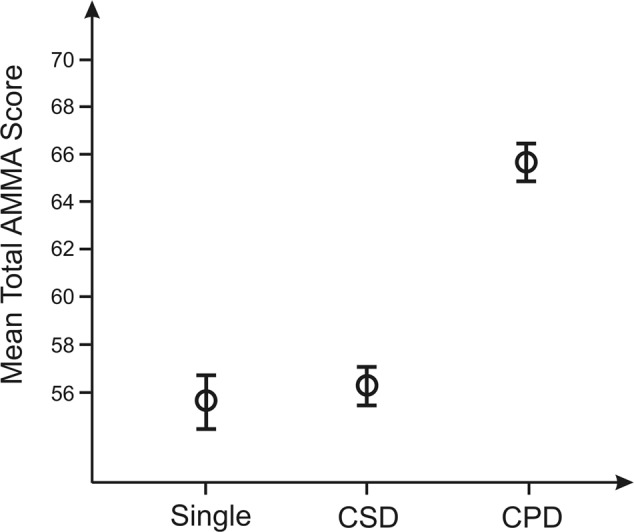
Results of the one-way ANOVA comparing mean total AMMA score with the three defined types of HG in the right hemisphere (for a visual presentation, see **Figure 1**). Error bars: SEM (standard error of the mean). Individuals with CPD had significantly higher scores in the AMMA test in comparison to subjects with SG or CSD in the right hemisphere.

Similar results were found for the AMMA test (**Figure 6**). The mean of the total AMMA score in the right hemisphere for SG was 55.7 ± 3.5, for CSD 56.5 ± 2.8, and for CPD 65.8 ± 2.8. Individuals with CPD achieved significantly higher scores than subjects with SG and CSD [*F*(2,26) = 3.8, *p* = 0.036, $\eta_{p}^{2}$ = 0.23]. There was no significant difference in the musicality test for SG and CSD.

These findings are also supported by the results of the PCA as presented in **Figure 2**. It is quite evident, that CPD are advantageous for all three components as revealed by the analysis. In other words, individuals with higher scores in the three components (i.e., musicality, language aptitude and working memory) also had more CPD.

## Discussion

The results of the principal component analysis, performed to gain insights into the most influential factors underlying performance on test scales, revealed three clear components, which very nicely reflect the three core aspects investigated in this study. They are (1) musicality (AMMA tonal, AMMA rhythm, number of instruments), (2) language aptitude (English score, all parts of the MLAT) and (3) working memory capacity (including the Hindi score and all working memory tests) (see **Table 3**). Since the main aim of our research was to explore the connection between these three variables and especially their relationship with auditory cortex morphology, they will be discussed separately in the upcoming paragraphs.

### Language Aptitude

Language aptitude is at the heart of our research and deserves sufficient attention with this regard. The Hindi score is the variable we assume to measure phonetic coding ability, i.e., a measure of a subcomponent of language aptitude. In the PCA, the Hindi score loaded on two components, namely working memory capacity and language aptitude. This nicely reflects the fact that working memory is an essential aspect of language aptitude and that the Hindi score can be seen as an indicator of both. Generally, the results of the PCA were very clear with regard to the component of language aptitude, which was clearly dominated by the MLAT, the English score and the Hindi score. This strongly supports their validity for measuring language aptitude.

The Hindi speech imitation task and the English pronunciation task measure very different components of language learning ability. The Hindi score is a speech imitation score, which requires the reproduction of unknown speech material. Still, it also demands the accurate auditory processing of this material. Otherwise, no successful imitation can take place. The English pronunciation task, in contrast, gives an overview of a subject’s pronunciation skills in their second language. Whereas the first is supposed to be a measure of phonetic coding ability (a major component of language aptitude), the second measures pronunciation proficiency in an already acquired language. High pronunciation proficiency, however, relies on a certain ability for phonetic coding and the two scores for language learning ability hence clearly go hand in hand. Even after correction for multiple comparisons, a positive, moderate correlation could be found between the two scores (*r* = 0.395, *p* = 0.031). The Hindi score could be seen as a precursor for the English score since high phonetic coding ability should lead to an excellent pronunciation in any language acquired by an individual. An issue with English in this case might be that many of the subjects had spent considerable time in an English-speaking country or had even studied English. English is a lingua franca and as education in Germany introduces English as first foreign language for every child, acquiring a native-like pronunciation is already supported from the beginning. Moreover, children are exposed to English in the early years of their lives, which might influence the success of their acquisition (critical periods are not well-defined but assumed to exist). This could explain why even individuals with lower scores in the Hindi test had a good pronunciation in English. We argue that frequent and long-lasting language exposure and contact are certainly reasons why the English scores were significantly higher. Since we see language aptitude as a predominantly innate capacity that unfolds over time in interaction with the environment, we assume that it is rather the Hindi score that should predict the English score, i.e., the better individuals are at decoding, retaining and reproducing unknown speech material, the easier it should be for them to develop excellent pronunciation skills in a given language.

Moreover, clear significant positive correlations were found between the English score and MLAT parts III and IV. The results in these two cases were quite robust, leading to two possible interpretations. First, the English score is related to an individual’s English skills and as the MLAT had to be used in English, it was probably more the subjects’ English proficiency that led to the high results. Second, high pronunciation proficiency in English is the result of particularly high language aptitude (phonetic coding ability and grammatical ability), as highlighted by the results of the correlational analysis. If we assume that the subtests of the MLAT, test III measuring phonetic ability and test IV measuring grammatical sensitivity, are excellent indicators of these components of language aptitude, we would at least expect a very high correlation between the Hindi speech imitation score and MLAT part III, which was not the case. But, as already mentioned, one of the major problems with the MLAT is that it is in English, giving individuals with better English skills a clear advantage over those subjects with less proficiency in English. Unfortunately, no German version of the MLAT exists to date, but in the past years language-independent tests, such as the LLAMA language aptitude battery (Meara, 2005), have gained popularity (Granena, 2013; Artieda and Muñoz, 2016; Rogers et al., 2016; Kepinska et al., 2017a,b). Therefore, only language-independent tests can exclude a possible influence of language experience and shall be used for future research. To get back to one aforementioned finding, English pronunciation skills also correlated with grammatical sensitivity (MLAT IV) and we assume that this should be rather a result of aptitude than of proficiency. Language analytic ability, the umbrella term under which grammatical sensitivity is nowadays subsumed, is an important component of language aptitude (Kepinska et al., 2017a,b) and should thus be of high significance for the learning of foreign languages. In our case, however, we focused on foreign language pronunciation ability (Jilka, 2009) and can only marginally address the significance of grammatical sensitivity. Looking at the last part of the MLAT, we see that vocabulary learning (part V – associative memory) did not correlate at all with the English score. It seems that vocabulary learning does not depend on proficiency but possibly on other factors such as learning strategies and motivation. Additionally, the claims that verbal working memory skills (attributed to the phonological loop) are essential for novel vocabulary learning (Gathercole and Baddeley, 1990; Atkins and Baddeley, 1998; Baddeley et al., 1998; Gathercole et al., 1999) could not be confirmed in our study, since MLAT V could not be linked to any working memory task.

One result that needs further discussion is the fact that the number of languages spoken by a subject did not show any relationship to any other score, except for age. It would be logical to assume that the more languages you speak, the better you are at learning different aspects of a new language (MLAT) or the better your English pronunciation and your speech imitation skills are. Vice versa, we would expect individuals with very high language learning ability to learn more languages due to the facility with which they acquire foreign languages. This was not the case due to various possible reasons. First, not everybody is willing to learn numerous languages for a number of reasons, e.g., a lack of time, opportunity or necessity. Secondly, having learnt a considerable number of foreign languages does not state anything about a subject’s proficiency or their learning process. The sample was limited but the number of foreign languages spoken by the individuals ranged from one to nine, which is quite an outstanding number. Another issue, however, is the fact that we were not able to control in any form how well the participants had learnt the languages and how well they were able to speak them at the time of the testing. It would have been necessary to include proficiency measures (grammar, vocabulary, and pronunciation) in all foreign languages in order to find the specific reasons why no correlations could be found. We further conclude that this result, i.e., that the number of languages does not impact an individual’s language aptitude, strongly supports our claim that language aptitude is a rather innate and inflexible capacity that cannot be altered through learning or practice at least. Although Eisenstein (1980) found that previous language training and bilingualism led to higher scores on the MLAT and also Thompson (2013) claimed that previous language experience may alter language aptitude, our study could not corroborate these findings. In the first case, this could also be explained due to excellent English skills. Since we assume, as already mentioned, that language aptitude is a trait somehow present before the acquisition of any language, it should not make a great difference whether an individual had learnt to speak three or nine languages. Earlier, language aptitude was seen as a stable construct that cannot be modified or developed through practice (Carroll, 1958 among others). Even though these assumptions have been questioned in the past decades (Klein, 1995; Sáfár and Kormos, 2008; Thompson, 2013), aptitude may not be as dynamic a construct as claimed by some researchers.

Other variables correlating positively and significantly with the Hindi speech imitation score were the number of instruments played by a subject and two tests of working memory capacity, namely digit span forward and non-word repetition. As has been discussed quite extensively in the introduction section, musical ability is very important for foreign language learning and playing an instrument certainly enhances auditory processing in an individual. For this reason, we expected a strong relationship between musicality scores (AMMA and number of instruments) with the Hindi speech imitation test (for details, see next paragraphs).

### Musicality

First, the PCA clearly defined three most influential factors for the musicality component, namely our musicality test (AMMA) and the number of instruments a subject played. It is noteworthy, however, that three other variables load on this component. The observation that non-word span also loads on the component musicality probably reflects the fact that musical processing builds upon temporally structured, but semantically undefined information that has to be kept in working memory. On the other hand, the fact that MLAT IV, which measures grammatical sensitivity, is moderately related to musicality, is only slightly surprising, as the understanding of language and music depends on internalized grammatical rules. It seems that the PCA factor musicality had a higher validity than the single subscales alone, which is evident from the fact that the AMMA scores and the number of instruments played were only positively but not significantly correlated with the number of languages spoken by subjects (see **Table 2**). The number of languages might thus be related to musicality on a more general level, which could not be sufficiently captured by the single tests.

Concerning musicality, the number of instruments played by an individual has often been assumed to have a considerable impact on a variety of cognitive skills, language acquisition just being one of them (Milovanov et al., 2008; Nardo and Reiterer, 2009). Music has very accurately been described as a resource that leads to auditory fitness (Kraus and Chandrasekaran, 2010) and positively influences the acquisition of skills in other domains, a phenomenon termed positive transfer. The correlations found in this study show a strong relationship between the musical domain and several language-relevant skills. Those who had very good scores in the language aptitude tests, also played more instruments and had better scores on the musicality tests. This supports findings from very recent research and confirms the strong relationship between the two (Milovanov et al., 2004, 2008, 2009, 2010; Magne et al., 2006; Dogil and Reiterer, 2009; Fonseca-Mora et al., 2011; Christiner and Reiterer, 2013; Seither-Preisler et al., 2014; Lee and Lin, 2015; Schön et al., 2004 among others).

Moreover, the number of instruments played by an individual also correlated positively and significantly with the Hindi score, which further confirms the expected strong relationship between music and language (Milovanov et al., 2008; Christiner and Reiterer, 2013; Seither-Preisler et al., 2014; Serrallach et al., 2016). In addition, the two parts of the AMMA correlated significantly with each other supporting the fact that people who have a certain musical ability are very good in different musical domains, in this case rhythmic and melodic discrimination abilities. The two AMMA parts further correlated positively and significantly with the number of instruments played showing that individuals who learn to play more instruments also have better auditory discrimination abilities, i.e., a functionally more efficient auditory cortex (Kraus and Chandrasekaran, 2010). The moderate correlation between the Hindi score and the AMMA test can be explained by the simple fact that the amount of time subjects had played the instruments and the amount of practice they had put into the learning process were not taken into account. These are definitely factors that need to be taken into consideration in future research.

Another interesting finding was the strong correlation between AMMA rhythm and English pronunciation skills. This was quite unexpected, in particular because no relationship with the Hindi score could be found. One option is that high rhythm skills and good rhythm perception facilitate the acquisition of a language and due to considerable practice and experience over time, this improves the participants’ pronunciation. In addition, the text used in this study, namely ‘The North Wind and the Sun,’ is very lyric-like (a fable for children, to be more precise) and may thus be associated with rhythmic perception or give an advantage to musically gifted individuals.

To conclude, for future research it will be important to spend more time investigating the concept of musical aptitude or musicality and using a variety of measures with the aim of fully grasping the construct. There are surely more factors that need to be taken into account and although most studies in this area use the AMMA test as a standard measure for musicality, it would be useful to additionally calculate an index of musical practice (see Seither-Preisler et al., 2014; Serrallach et al., 2016). This provides a fine-grained measure for musical expertise and it allows the implementation of numerous aspects of musicality (different music-related skills and associated variables such as amount of practice, singing interest etc.). This will surely be of high relevance when further investigating the relationship between language and music.

### Working Memory Capacity

It does not come as a surprise that speech imitation requires excellent working memory skills and the claim that working memory makes up quite a considerable part of language aptitude is surely not far-fetched (Miyake and Friedman, 1998; Wen and Skehan, 2011; Wen, 2012; Wen et al., 2017). Other studies that have challenged this assumption found that speech imitation skills rely heavily on working memory (Ellis and Sinclair, 1996; Miyake and Friedman, 1998; Kormos and Sáfár, 2008; Sáfár and Kormos, 2008; Biedroń, 2012; Linck et al., 2013). Consistently, we found a positive relationship between speech imitation skills and different measures of working memory, as indicated in all studies above. The three measures we applied were digit span forward, backward, and non-word span. Two of them correlated positively and significantly with the Hindi score, the non-word span showing the highest correlation with this respect. It was only the Hindi test and the age of participants, that showed a strong relationship to non-word span, however. It is common knowledge that children cannot be compared to adults with regard to measures of working memory since working memory seems to improve over time. In this study, this could only be confirmed for non-word span and not for the other two working memory tasks. It seems questionable that working memory capacity improves with age in individuals between 20 and 40 years. Rather, we assume that it might be that the older participants were also those with generally better non-word span, which led to the finding.

In the correlational analysis, the three working memory scores could not be linked to any other variable. The PCA, in contrast, showed a clearly defined component for working memory capacity, based on test results in the Hindi testing, non-word span, digit span forward and backward. In this analysis, the number of instruments loaded on this component as well, supporting a possible influence between musical expertise and high working memory capacity. Moreover, the PCA also points toward a stronger relationship between the three working memory scores, and also with the Hindi score, than shown in the correlational analysis.

One of the main assumptions of this study was that phonetic coding ability is the component of language aptitude that should be best measured through the Hindi test. However, it seems that the non-word span captures a very similar ability. Furthermore, the non-word test has also been used as an indicator for specific language impairment (Botting and Conti-Ramsden, 2001; Coady and Evans, 2008), supporting our hypothesis of it being a test measuring high or particularly low language learning ability. The Hindi test requires the decoding of unfamiliar speech, retaining it for a particular amount of time and the ability to reproduce it as correctly as possible. Despite some slight differences, both the non-word task and the Hindi speech imitation task use speech material that basically consists of simple CV (consonant-vowel) syllables. So whereas the working memory load increases in the non-word span, the same syllables have to be repeated only adding one element at a time. In the Hindi task, the words and sentences always change and something completely new has to be reproduced. In sum, both heavily rely on working memory capacity and we propose that both tests are equally significant and useful measures of working memory capacity on the one hand, and phonetic coding ability on the other hand. We therefore propose to further develop non-word tests in order to ameliorate language aptitude testing batteries (Chan et al., 2011).

Digit span forward also correlated positively and significantly with the Hindi score, but not with any other score. This is a little surprising because other studies (Van den Noort et al., 2006; Kormos and Sáfár, 2008; Biedroń, 2012) have shown that high ability and success in foreign language acquisition, in our case the English score, correlate with working memory tasks of differing complexity. Nevertheless, considering that the Hindi score is our main language aptitude score, we conclude that both simple and complex working memory skills are required to imitate foreign speech, i.e., for phonetic coding ability. This further supports the hypothesis that working memory is a core component of foreign language aptitude (Wen, 2016; Wen et al., 2017) but we do not agree with the hypothesis that working memory may be seen as an equivalent to language aptitude (Linck et al., 2014).

Last but not least, one surprising finding is a lack of relationship between the three working memory tests. The construct of working memory includes different components, which are expected to influence one another or at least share some common basis. Our study, however, did not show any correlation between the three scores. Only the PCA showed the dependence between the three variables and also the Hindi testing. We therefore propose that different components of working memory are indeed relevant for language learning but to a certain extent independent from each other.

### Neuroanatomic Markers for Language Aptitude and Musicality

Studies of the past years have partly investigated the neural basis of language learning ability and they have certainly highlighted the significance of the structure of language-relevant regions in the human brain. As Berken et al. (2015) correctly summarizes, structural variation in the brain can indeed reveal variation in language aptitude. Learning novel elements of a language, e.g., tonal pitch contrasts and phonetic differences (Golestani and Zatorre, 2009), and perceiving and producing novel speech sounds (Golestani et al., 2002; Golestani and Pallier, 2007) can reveal interesting information as to which regions are important for these processes. Mostly, language has been ascribed to the left hemisphere and also findings regarding HG (with respect to language) have emphasized the special role of the left side (Golestani and Pallier, 2007; Golestani et al., 2007; see Introduction).

The most interesting finding of the neuroanatomic analysis of this study is that individuals with high Hindi scores also had more CPDs of HG in the right hemisphere, contradicting theories of leftward lateralization for language functions in healthy adults. In this regard it is revealing that also the AMMA score showed a particularly strong relationship with HG duplications in the right hemisphere. Evidently, both skills, even if not being directly correlated with each other, appear to be closely linked to right HG. Most interestingly, when the participants’ individual factor scores on each of the three components were compared for the three HG types, it became obvious that a CPD seems to be correlated with high results in all three components of the PCA (see **Figure 2**). In other words, for all three factors defined (musicality, working memory, and language aptitude), performance of subjects with CPD was highest.

There are two main topics that need to be discussed accordingly with respect to these findings. First of all, the results suggest more than just a positive relationship between language aptitude, music and partly also working memory. It is necessary to specify the nature of this relationship, the influence of the auditory cortex on the two and why it is only the right hemisphere that seems to be much more important. Second, the function of CPDs in HGs is far from being clear. Leaving aside the hemispheric differences, there is convincing evidence for a specific structure-function relationship of HG duplications and furthermore a considerably larger prevalence of HG duplications in both musicians (Benner et al., 2017) and linguistically talented subjects (Golestani et al., 2011). The connectivity between the first HG, hosting in most cases the primary auditory core areas, and the different HG duplications, hosting among others associated language-related belt and parabelt areas, may have a hitherto unknown impact on auditory functioning and thus the development of language and musical skills.

Since our main aim was to find the neuroanatomical markers of language aptitude and language has been claimed to be predominantly left-lateralized, we expected to find differences mainly in the left hemisphere (Golestani et al., 2002, 2007, 2011; Golestani and Pallier, 2007; Wong et al., 2007; Dogil and Reiterer, 2009; Golestani and Zatorre, 2009; Reiterer et al., 2011; Warrier et al., 2012; Hu et al., 2013). Yet, in the current study the occurrence of duplications in the left hemisphere was considerably lower as compared to the balanced distribution in the right hemisphere (see **Table 3**). Given the little variation found in the left hemisphere, a much larger sample would have been needed to perform corresponding group statistical comparisons for the left hemisphere. Therefore, all further discussion focuses on the right hemisphere only.

It is well-known that musical ability heavily relies on the right hemisphere and also recent research has shown the significance of HG on the right side for musical processing. But why does HG in the right hemisphere relate so well with the Hindi testing then? There are numerous possible explanations. To put it simple, the results clearly indicate that the shape of auditory cortex and the number of HGs in the right hemisphere are linked to musical and linguistic ability. Relationships with working memory, in contrast, could only be found through the PCA, in which the component of working memory capacity included the Hindi score. Therefore, working memory and AC structure will not be discussed in detail here. To get back to the main issue, only the individuals with a CPD (i.e., two complete gyri) in the right hemisphere had significantly higher scores in the AMMA test and in the Hindi task. The individuals with single gyri and CSDs (not counted as two complete gyri) had substantially lower results in both. Is it therefore necessary to have two HGs in the right hemisphere to have a considerable advantage in auditory processing? And if we assume that individuals with a double HG have a functionally advantageous auditory cortex, why is it that both language and music seem to be so heavily influenced by it?

In a larger sample we might have discovered even more robust evidence for the observed relationship between language aptitude and musicality. Other studies, however, have already shown that musical ability facilitates language learning. The Hindi speech imitation task is basically a working memory capacity task that requires good use of the articulators to produce foreign speech material and a functionally efficient auditory cortex to hear the subtle differences in the speech input. Could it be that only phonetic coding ability, i.e., only this particular component of language aptitude, is highly dependent on (1) musical ability or (2) the processing of auditory cortex in the right hemisphere? If phonetic coding ability were dependent on auditory processing of music-relevant features in the right hemisphere, this would explain why only the right hemisphere showed double gyri in most subjects. One of the most difficult questions in this regard is to what extent differences in auditory cortex are due to language aptitude or due to musical ability. It could be that, as in other studies, we just found a confirmation of the importance of CPDs in the right hemisphere for musical processing and due to the fact that Hindi speech imitation requires non-speech processing expertise, we found a similar relationship between the two. Another possibility is that we found a neuroanatomical marker for foreign language pronunciation aptitude and this marker also influences musical processing, leading to a high capacity in both domains. Third, our results could suggest that the right hemisphere is more important than assumed for elementary auditory processing, which is at the basis of both speech and music. Even though we do not doubt that numerous areas in the brain are of high importance for the processing of language, our results clearly highlight the significance of auditory cortex as an essential area of auditory processing.

It could be that AMMA and Hindi, which did not show a direct correlation in this study, are independently linked to right hemispheric functions that require more HGs for efficient auditory processing. Other studies have shown that the individual morphology of these structures, despite high inter-individual variation, are extremely stable from childhood into adulthood (Seither-Preisler et al., 2014). It could thus be assumed that individual differences are first and foremost not due to environmental influences or practicing behavior. Rather, they appear to have a strong biological component, which may be genetic, prenatal, or very early post-natal. As yet, it is also unclear of how the gross-morphological structural characteristics of auditory cortex are related to characteristic functional activation patterns. This important aspect should be specifically addressed in subsequent investigations. In particular, the kind of advantage a CPD of HG has in an individual’s brain and if and how this affects language learning and musical ability remains to be uncovered. In addition, even if we know that traits such as shape and number of gyri in auditory cortex play a certain role, it cannot be deduced what kind of advantage CPDs give an individual for language or music processing.

We are aware that the view of language aptitude has changed in the past decades and it is more and more frequently referred to as a dynamic construct that may indeed undergo change over time. Quite recently, an appealing study by Kepinska et al. (2017a,b) on language analytic ability highlighted the significance of the right hemisphere for language aptitude. Moreover, Prat et al. (2016) reported right-hemispheric involvement among highly successful L2 learners in their resting-state qEEG study with adult learners. The right hemisphere might thus be more important for language acquisition and processing than initially assumed. More research will be needed to explore the involvement of the right hemisphere, in particular the right HG, in different aspects of language aptitude. Also, given the various regions in the brain that are essential for language processing, we will aim at developing methods in order to structurally analyze other significant areas, such as the inferior parietal lobule (Dogil and Reiterer, 2009; Reiterer et al., 2011; Hu et al., 2013; Golestani et al., 2002, 2007, 2011 among others).

To sum up – if it is possible to determine neuroanatomical markers that remain highly stable from early infancy to adulthood, this challenges the assumption that the capacity to acquire associated behavioral skills can be substantially altered throughout lifetime. Furthermore, if the structures of certain brain regions are strongly related to specific behavioral skills, we have to find out how they control the natural unfolding and development of these skills. Although there is no doubt that numerous external variables also influence the development of language and musical skills, we support the claim that there are strong innate and/or prenatally determined neurological factors that remain to be uncovered in the next decades. We are already working on similar investigations in children with differing degrees of musicality and language ability in order to confirm and extend the results of this study. We would also like to encourage other researchers to investigate language aptitude from an anatomical viewpoint, additionally to functional differences that have been repeatedly found in individuals with high and low language learning ability.

## Ethics Statement

This study was carried out in accordance with the recommendations of ‘Ethikkommission des Universitätsk linikums Tübingen (Germany)’ with written informed consent from all subjects. All subjects gave written informed consent in accordance with the Declaration of Helsinki. The protocol was approved by the ‘Ethikkommission des Universitätsklinikums Tübingen.’

## Author Contributions

All authors contributed to the submitted paper. ST was responsible for the design of the work, data analysis and interpretation and drafting the article. SR contributed extensively to the data collection. All authors, PS, AS-P, SR, and ST performed a critical revision of the paper and gave their approvement to the final version for submission.

## Conflict of Interest Statement

The authors declare that the research was conducted in the absence of any commercial or financial relationships that could be construed as a potential conflict of interest. The reviewer OK and handling Editor declared their shared affiliation.
